# Editorial: Climate and health education: defining the needs of society in a changing climate

**DOI:** 10.3389/fpubh.2023.1307614

**Published:** 2023-10-27

**Authors:** James K. Sullivan, Gaurab Basu, Lisa Patel, Arianne Teherani, Cecilia Sorensen

**Affiliations:** ^1^Global Consortium on Climate and Health Education, Mailman School of Public Health, Columbia University, New York, NY, United States; ^2^Cleveland Clinic Lerner College of Medicine, Cleveland, OH, United States; ^3^Center for Climate Health and the Global Environment, Harvard T.H. Chan School of Medicine, Boston, MA, United States; ^4^Medical Society Consortium on Climate and Health, George Mason University, Fairfax, VA, United States; ^5^Stanford School of Medicine, Stanford University, Stanford, CA, United States; ^6^University of California Center for Climate, Health and Equity, University of California, San Francisco, San Francisco, CA, United States; ^7^Department of Emergency Medicine, Columbia Irving Medical Center, New York, NY, United States; ^8^Department of Environmental Health Sciences, Mailman School of Public Health, Columbia University, New York, NY, United States

**Keywords:** climate and health, climate and health education, health professional competencies, environmental education, climate and society, climate change

The effects of climate change due to the burning of fossil fuels are apparent and present increasingly complex challenges to human health (1). Climate change poses direct health risks, including extreme weather events, and indirect risks, such as long-term ecological changes leading to changes in air quality or vector habitats that alter patterns of infectious disease (2–9). The changing climate also poses diffuse and deferred risks because of long-term societal changes, civil conflict, and disrupted livelihoods leading to mental or physical health effects and refugee displacement (10, 11). Healthcare delivery is jeopardized either through barriers to community access or hospital operations via supply chain issues and extreme weather events (12–14). These impacts intertwine structural racism and environmental injustice that result in poor communities, communities of color, and communities in the global south being impacted disproportionately. With many complex, intersectional, and transdisciplinary challenges, there is an urgent need to train health professionals across disciplines, yet most health professionals have not been trained and climate and health curricula are nascent in programs around the world (15).

We need to build health professional workforce capacity to understand the risks of climate change, what they as providers can do to help patients mitigate and adapt, and how to be changemakers in health systems. Climate-smart health professionals need a nuanced understanding of the intersectionality of inequity, structural racism, and other social determinants of health. Further, these needs do not exist in a vacuum; building capacity to train climate-smart professionals is also an opportunity to provide clinical, policy, education, and advocacy career paths to help address the innumerable intersectional challenges adversely affecting patients around the globe.

The recent series from *Frontiers in Public Health, “Climate and health education: fefining the needs of society in a changing climate”* provided an opportunity for many educators around the world to showcase their critical work addressing these challenges. The work described in this series sets the context for the current direction of climate change and health education and begins to point the path forward on areas for future work. Some manuscripts described the rapidly evolving state of climate and health education. Houghton reviewed 99 courses at 3 different United States universities covering issues related to climate change, health, and equity in the built environment. Though they found more courses covering these topics than prior analyses, too often the content was isolated. There is a need to explicitly connect population health, the built environment, and climate change with transdisciplinary content as the built environment plays a leading role in creating the context that drives disparities in population health and is a substrate for exacerbating inequity in the climate crisis. Arora et al. found that half of public health schools in the United States offered at least one climate change related course and half of climate change courses specifically covered health impacts. Simon et al. performed a qualitative analysis of medical school stakeholders in Germany, highlighting a high prevalence of positively reviewed climate curricula, but unmet needs in transdisciplinary education, incorporation of ethics, and practical skill training, such as patient communication and physical diagnosis.

Other manuscripts detailed initiatives that centered on student-faculty co-creation of content related to climate and health. Navarrete-Welton et al. demonstrated the power of a student driven, bottom-up approach to build an integrated, broad-reaching curriculum at a United States medical school that not only covers health effects related to climate, but also built capacity to train students to be changemakers with a dedicated course on waste management in healthcare. Liu et al. performed a qualitative analysis of medical students completing climate and health curricula, uncovering a desire for more small group learning, clinical skills integration, and community-based opportunities. Along with the student perspectives described by Simon et al., it is clear that while students positively perceive climate and planetary health initiatives, there is a need to connect these topics to additional societal issues taught in schools.

Lastly, several works described projects at the intersection of leadership, accountability, and communication that point to how healthcare and public health professionals might help mitigate, adapt, and respond to the climate crisis. Dambre et al. performed a qualitative analysis of focus groups of undergraduates in a Global Responsibility and Leadership program at a Netherlands University that participated in a planetary health course. Their course received high marks for transdisciplinary integration of climate, health, and communication, but the major unmet need was transcultural content. Schmeltz and Ganesh highlighted student-led collaborations with local organizations, demonstrating how undergraduate students can be part of capacity building initiatives. Lastly, Campbell et al. reviewed current research on climate and health communication strategies and highlighted evidence for health-based messaging to increase engagement and political will for climate solutions, in addition to evidence for naming the role of fossil fuels when discussing climate change.

This series from *Frontiers in Public Health* highlights the efforts of educators and students around the globe to rapidly innovate and train the next generation of health professionals to be equipped to treat patients, build capacity, and advocate for essential societal change to confront the climate crisis. This field is expanding rapidly. For example, after a recent burst of new curricular development, over 50% of US medical schools now include climate-related topics (16), though integrated curricula are more limited (17). Longitudinal integration of climate and health touchpoints in multiple existing curricular activities is necessary for students to develop a climate and health lens to incorporate climate into their future health practice (Liu et al.) (17–20). Further, existing climate curricula could benefit from more transdisciplinary and community-oriented approaches. Partnerships with environmental justice organizations, pairing students with community organizations to learn directly from stakeholders, and involving collaborators from disciplines beyond healthcare are just a few possible solutions to help break down the silos and paradigms of existing educational approaches that can inadequately prepare students to partner with communities. We need more institutions and individuals to innovate, evaluate, and disseminate longitudinal, integrated training programs that enable the next generation to fill these roles.

## Author contributions

JS: Writing—original draft, Writing—review & editing. GB: Conceptualization, Writing—review & editing. LP: Conceptualization, Writing—review & editing. AT: Conceptualization, Writing—review & editing. CS: Conceptualization, Supervision, Writing—original draft, Writing—review & editing.
